# Exploring the landscape of artificial intelligence in dental and maxillofacial radiology: a bibliometric analysis of studies and trends

**DOI:** 10.1097/MS9.0000000000005013

**Published:** 2026-06-02

**Authors:** Mohammad Amin Amiri, Hariprasad Reddy Korsapati, Gokhan Anil, Abinash Mahapatro, Abdulhadi Alotaibi, Farahnaz Joukar, Fariborz Mansour-Ghanaei, Soheil Hassanipour, Mohammad-Hossein Keivanlou, Reza Delbari, Amirreza Hendi, Mahsa Koochaki

**Affiliations:** a Oral and Dental Disease Research Center, Shiraz University of Medical Sciences, Shiraz, Iran; b 5-Mayo Clinic Health System, 1025 Marsh Street, Mankato, MN, USA; c SIU School of Medicine, Springfield, IL, USA; d Department of Medicine, Vision Colleges, Riyadh, Saudi Arabia; e Gastrointestinal and Liver Diseases Research Center, Guilan University of Medical Sciences, Rasht, Iran; f School of Medicine, Guilan University of Medical Sciences, Rasht, Iran; g Dental Sciences Research Center, Department of Prosthodontics, School of Dentistry, Guilan University of Medical Sciences, Rasht, Iran; h Dental Sciences Research Center, Department of Oral and Maxillofacial Medicine, School of Dentistry, Guilan University of Medical Sciences, Rasht, Iran

**Keywords:** artificial intelligence, deep learning, dental imaging, machine learning, maxillofacial radiology

## Abstract

**Background:**

The integration of artificial intelligence (AI) in dental and maxillofacial radiology is revolutionizing diagnostic accuracy and clinical decision-making. This bibliometric analysis investigates the research landscape, emerging trends, and scholarly impact of AI applications in this specialized field.

**Methods:**

A comprehensive search was conducted on 25 December 2024 using the Web of Science Core Collection database. Data analysis tools, including VOSviewer, CiteSpace, and Biblioshiny, were employed to examine publication trends, global contributions, collaborative networks, and keyword dynamics.

**Results:**

The analysis revealed a marked increase in AI-related publications in dental and maxillofacial radiology, particularly from 2016 onward. The number of studies rose steadily, reaching 218 publications in 2024. The United States led in research output, followed closely by China and South Korea, with KU Leuven emerging as the top-contributing institution. Reinhilde Jacobs was identified as the most prolific author, while Medical Physics was the most cited journal. Co-citation analysis highlighted influential works by authors such as J.H. Lee and F. Schwendicke . Keywords including “artificial intelligence,” “deep learning,” “CBCT,” and “classification” dominated research discussions, reflecting the field’s evolving focus. Recent research trends emphasize advanced applications in segmentation, accuracy enhancement, and predictive modeling.

**Conclusion:**

AI has become integral to the advancement of dental and maxillofacial radiology, offering significant improvements in diagnostic precision and treatment planning. This study underscores the importance of staying abreast of AI innovations to enhance patient care and foster future research opportunities. Researchers and clinicians are encouraged to adopt AI-driven approaches to maximize clinical efficiency and outcomes.

## Introduction

The application of artificial intelligence (AI) in medicine has emerged as a transformative paradigm, offering innovative solutions to address persistent challenges across multiple healthcare disciplines^[^1–4^]^. Among these, dental and maxillofacial radiology has gained particular attention as a field well suited for AI integration due to its reliance on complex image-based data for diagnosis and treatment planning^[^5–7^]^. Recent advances in AI-driven methodologies have initiated a paradigm shift in this specialty, with increasing exploration of their potential for routine clinical implementation^[^8–10^]^.

HIGHLIGHTS

- Comprehensive analysis of artificial intelligence (AI) in dental and maxillofacial radiology highlights its transformative role in improving diagnostic accuracy and clinical decision-making.
- Exponential growth in AI-related research since 2016, with emerging trends in CBCT and deep learning, reflects the field’s evolving focus on advanced imaging techniques.
- Keywords like “deep learning,” “CBCT,” and “segmentation” emphasize the focus on diagnostic precision, while co-citation analysis reveals critical contributions shaping the field’s development.

Dental and maxillofacial radiology encompasses a broad spectrum of imaging modalities, including cone-beam computed tomography (CBCT), computed tomography (CT), periapical, bitewing, panoramic, and occlusal radiography, as well as sonography and magnetic resonance imaging. They provide complementary visualization of hard and soft tissues, enabling comprehensive assessment of the maxillofacial region for diagnostic and therapeutic purposes^[^11–13^]^. The development of precise and robust computational models capable of quantitatively and qualitatively analyzing anatomical structures across these imaging modalities has the potential to significantly enhance diagnostic accuracy and optimize treatment planning^[^14–16^]^.

AI-based models, particularly those employing deep learning techniques, have demonstrated exceptional performance in identifying complex patterns and subtle imaging features that may be challenging for human observers^[^17–20^]^. In dental and maxillofacial radiology, these algorithms have been increasingly applied to tasks such as image classification, anatomical structure segmentation, lesion detection, anomaly identification, and predictive modeling. Such applications show promise in improving diagnostic efficiency, reducing inter- and intra-observer variability, and supporting clinical decision-making^[^6,21–24^]^.

The marked increase in AI-related publications in dental and maxillofacial radiology over recent years reflects growing scientific interest and investment, driven by advances in computational power, data availability, and imaging technologies^[^25–27^]^. In this context, the present bibliometric analysis aims to provide an overview of the evolving research landscape, identify influential contributors and thematic trends, and highlight emerging directions for the effective integration of AI into dental and maxillofacial radiology.

## Methods

On 25 December 2024, a comprehensive literature search was conducted using the Web of Science Core Collection as the principal database. The Web of Science Core Collection is recognized for its broad scope, covering more than 12 000 reputable journals. To enhance the effectiveness of the search, a multifaceted search strategy (outlined in Supplemental Digital Content Table S1, available at: http://links.lww.com/MS9/B209) was employed. Initially, 780 records were retrieved. After removing conference proceedings, letters, editorials, book chapters, pre-publication items, duplicates, and studies that did not meet the research objectives, a total of 751 relevant papers remained. Only original research and review articles were included. The decision to include only original research and review articles stemmed from their rigorous peer-review processes, which ensure both credibility and scientific integrity. Other types of literature, such as conference proceedings, editorials, and books, were excluded due to their typically less stringent peer-review and indexing practices, which may undermine the reliability and consistency of citation patterns in bibliometric studies. Furthermore, retracted papers were eliminated to preserve the dataset’s accuracy, as these do not reflect trustworthy scientific contributions. Non-English publications were also omitted because the bibliometric tools employed are specifically designed for English-language texts. By focusing solely on English papers, we ensured consistency in the analysis, as these tools are not fully effective with non-English materials. Study selection was conducted independently by two researchers based on predefined inclusion and exclusion criteria. Any disagreements regarding study eligibility were resolved through discussion and consensus.

## Data analysis

All relevant documents were converted into Microsoft Excel 2019 and plain text formats to facilitate their analysis using Biblioshiny, VOSviewer, and CiteSpace. VOSviewer is recognized as a robust tool for scientometric network analysis, helping clarify how academic literature is interconnected through visual representations and network-based maps. By harnessing co-citation, co-occurrence, citation, and bibliographic coupling relationships, it generates network diagrams that depict various scholarly elements, including publications, journals, authors, research institutions, countries, and keywords^[^28–30^]^.

CiteSpace, a Java-based citation visualization tool, employs advanced data mining, extensive information processing, and comprehensive knowledge mapping. This software showcases the evolution, distribution patterns, and structural elements of scientific knowledge. By visually representing citation networks, CiteSpace highlights significant nodes and emerging trends, offering insights into the shifting context of scientific research^[^31–33^]^.

Biblioshiny is a user-friendly graphical interface for the R Bibliometrix package. It offers a comprehensive suite of tools for bibliometric analysis, allowing users to conduct citation analysis to assess the influence of papers, authors, and institutions. Additionally, it facilitates co-authorship analysis to investigate collaboration patterns within networks^[^34–38^]^.

## Results

### Publication trend

**Figure 1.**
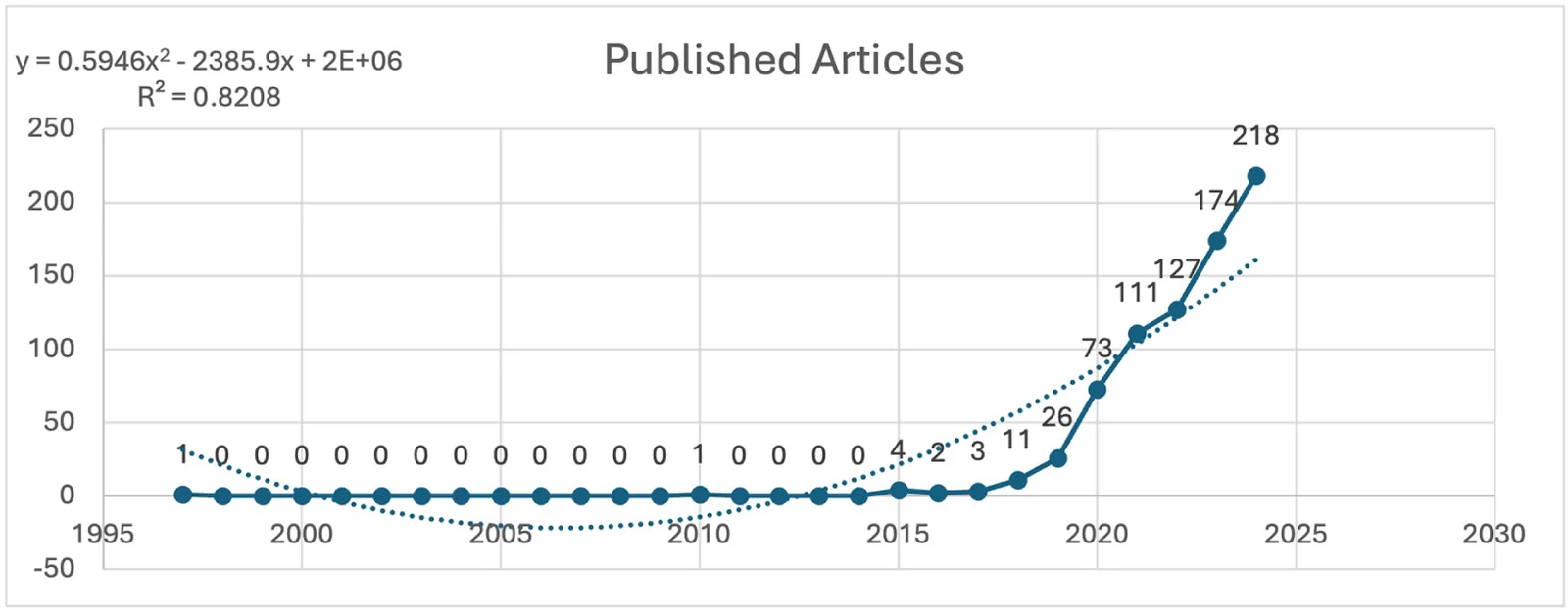
Annual publication trends on artificial intelligence in dental and maxillofacial radiology. This figure illustrates the yearly growth in published studies, highlighting the rapid increase in research activity.

**Figure 2.**
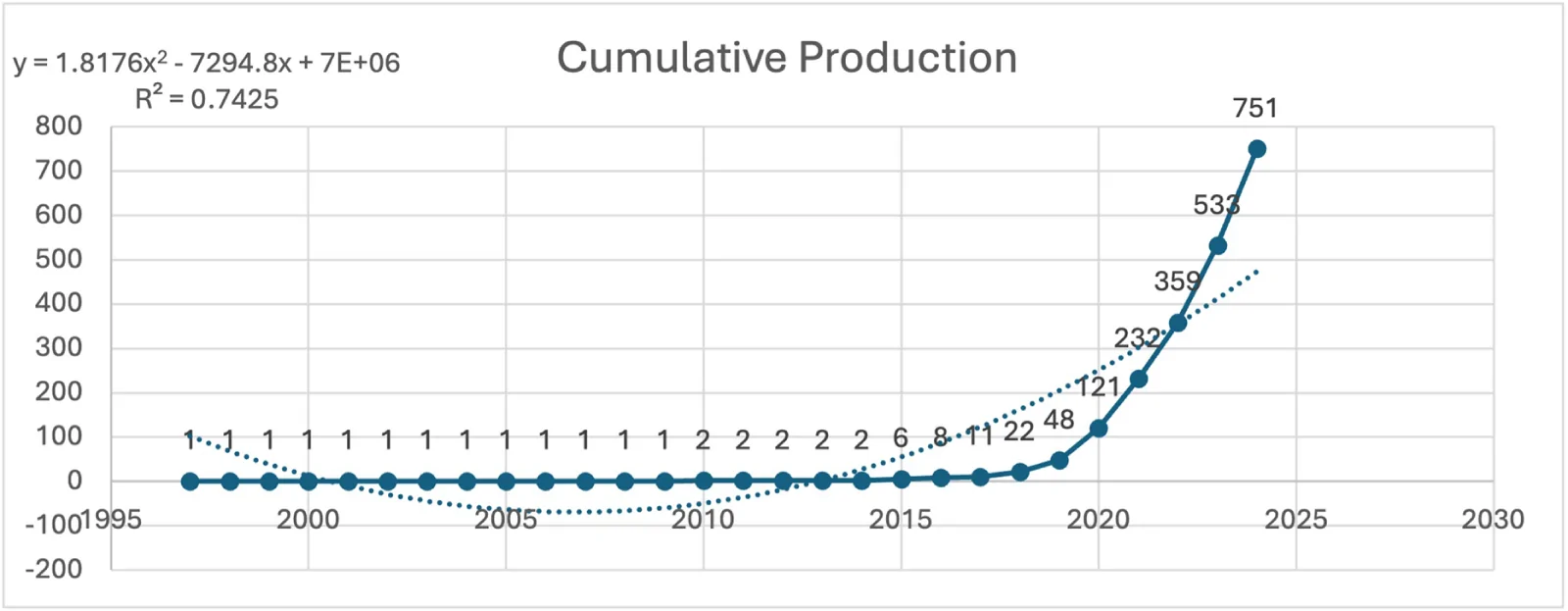
Cumulative publications on artificial intelligence in dental and maxillofacial radiology. This graph shows the total number of publications over time, reflecting the accelerating growth in research output.

Trends in research within a specific field are often revealed by examining the number of publications produced over time. In the early stages, research on AI in dental imaging was minimal, with very few publications between 1997 and 2015. For instance, from 1997 to 2015, only six papers were published, and the cumulative output remained stagnant at one publication until 2010. However, starting in 2016, there was an increase in research activity. Between 2016 and 2017, the number of publications reached a modest total of 11, signaling a growing interest in the field. A surge occurred from 2018 onward, with the number of published articles rising sharply. In 2018, 11 papers were published, followed by 26 in 2019, and a major jump to 73 in 2020. This upward trend continued, with 111 publications in 2021 and 127 in 2022. By 2023, the number of publications had risen to 174, and in 2024, it reached a peak of 218 publications (Fig. 1). The cumulative production over time shows a steady upward trend, particularly after 2015. From a total of just 11 cumulative publications by 2017, the field grew further, reaching 533 cumulative papers by 2023 and 751 by 2024. This rise highlights the growing impact and importance of AI within the realm of dental imaging (Fig. 2).

### Countries and institutions

**Figure 3.**
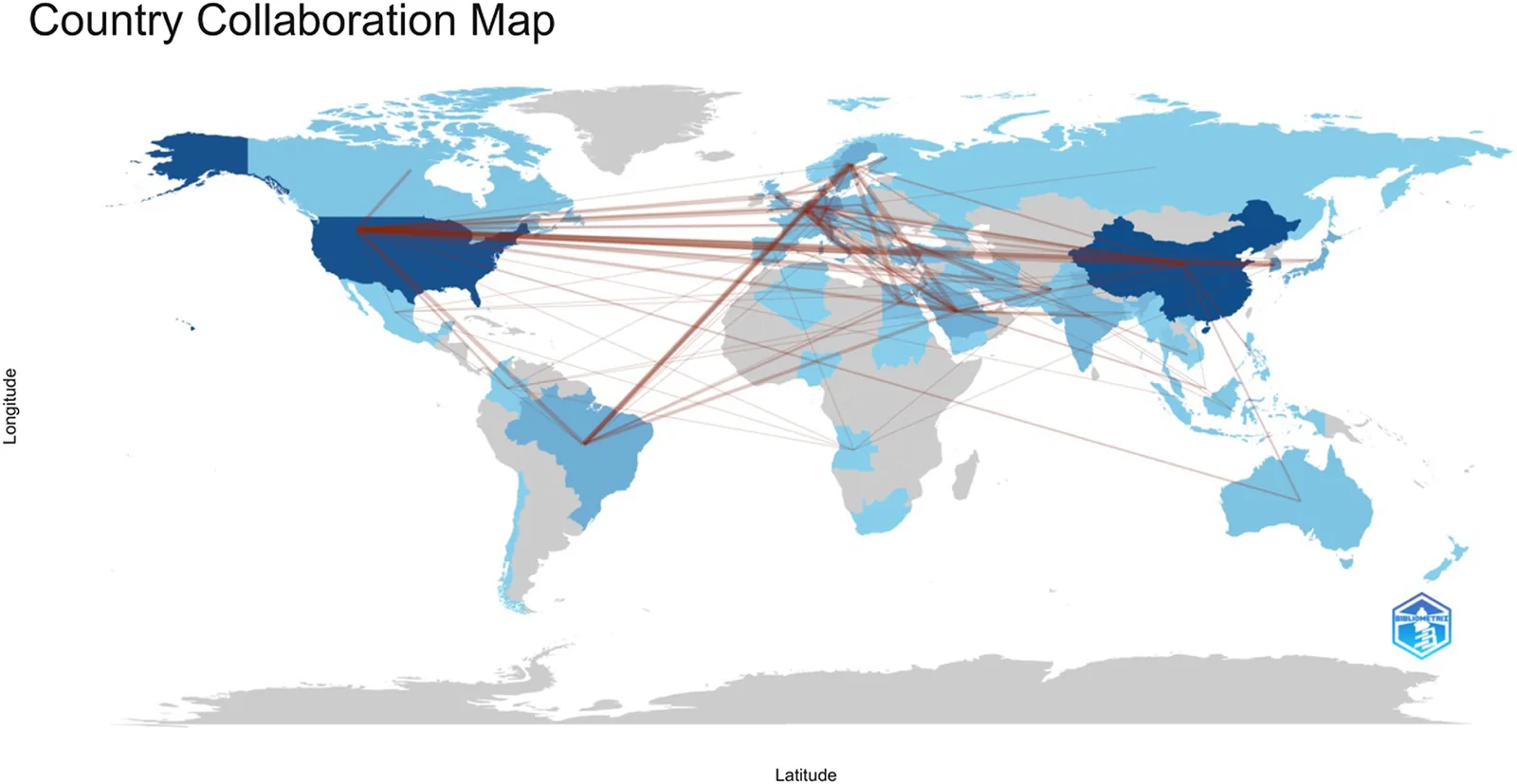
International collaboration network in AI-related dental imaging research. The figure depicts country-level collaborations, highlighting the global nature of research efforts in this field.

The analysis of global contributions showed that 94 countries participated in collaborations within this research field (Fig. 3). The analysis of the top 10 countries by publication output revealed that the United States led with a substantial contribution (*n* = 166), followed closely by China (*n* = 161). South Korea ranked third with publications (*n* = 75), followed by Germany (*n* = 64), Belgium (*n* = 49), Sweden (*n* = 44), Brazil (*n* = 41), Saudi Arabia (*n* = 40), Japan (*n* = 40), and the Netherlands (*n* = 38), each making notable contributions to the field (Table 1).

**Table 1:** Top 10 countries by the number of publications.

Rank	Country	Number of publications
1	USA	166
2	People’s Republic of China	161
3	South Korea	75
4	Germany	64
5	Belgium	49
6	Sweden	44
7	Brazil	41
8	Saudi Arabia	40
9	Japan	40
10	Netherlands	38

**Figure 4.**
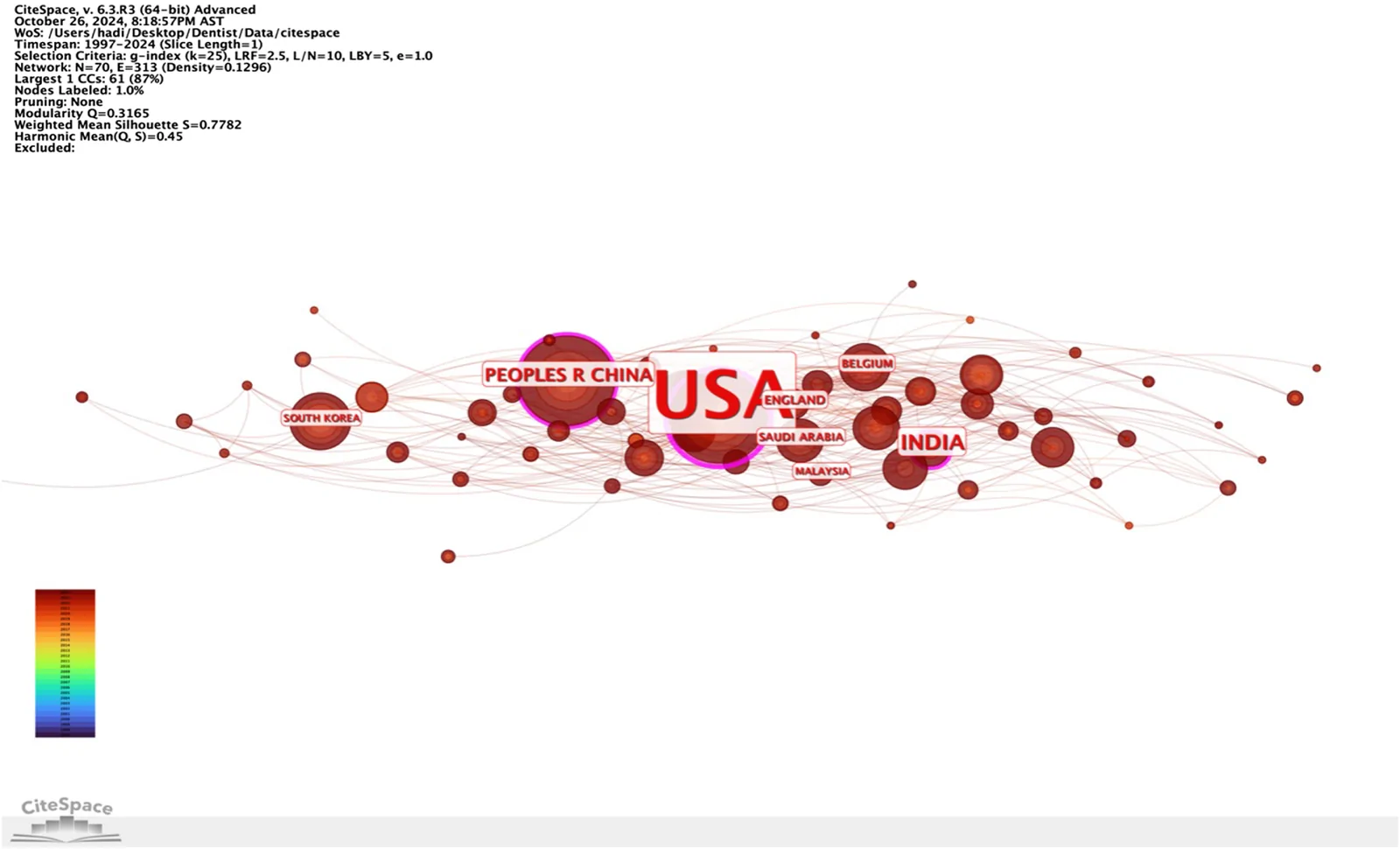
Countries with the highest centrality in collaborative research networks. This map identifies countries that act as key hubs in the global research network, based on centrality measures.

**Figure 5.**
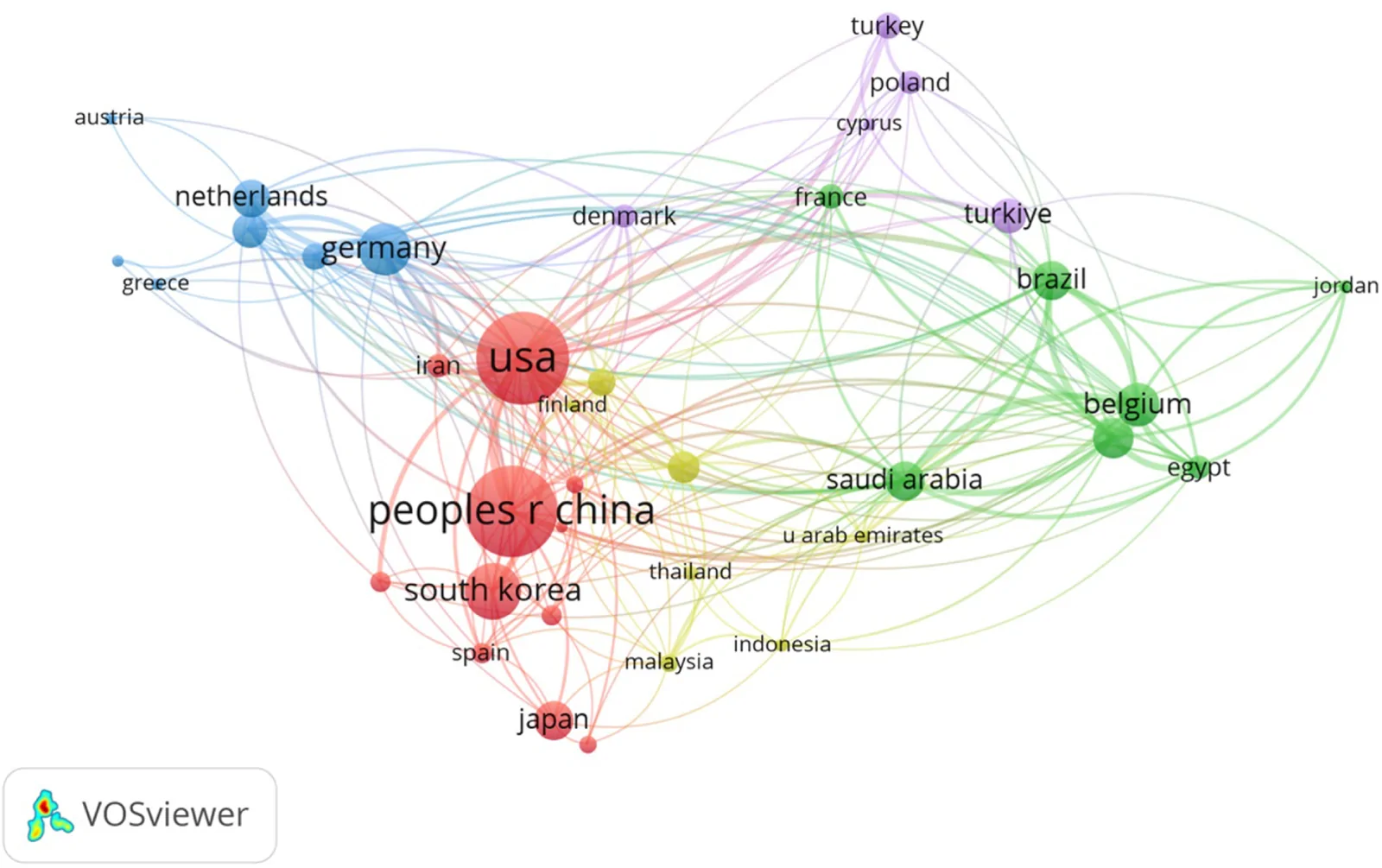
Network visualization of country-level collaboration in AI-based dental imaging research. The visual illustrates the strength and structure of international research ties among participating countries.

The top 10 countries in terms of centrality are presented in Table 2. The United States emerged as the country with the highest centrality (0.45), followed by India (0.15). Other countries with notable centrality values included China (0.12), Belgium (0.08), Saudi Arabia (0.08), and England (0.08). South Korea (0.07), Malaysia (0.07), Germany (0.06), and the United Arab Emirates (0.05) also played critical roles in maintaining the cohesion and influence of the research network (Table 2). Figure 4 illustrates the collaborative network among countries, highlighting central nodes like the USA and India, which served as major hubs of international collaboration. Figure 5 shows the strength of collaborative ties between different countries.

**Table 2:** Top 10 countries by centrality.

Rank	Country	Centrality
1	USA	0.45
2	India	0.15
3	People’s Republic of China	0.12
4	Belgium	0.08
5	Saudi Arabia	0.08
6	England	0.08
7	South Korea	0.07
8	Malaysia	0.07
9	Germany	0.06
10	United Arab Emirates	0.05

**Figure 6.**
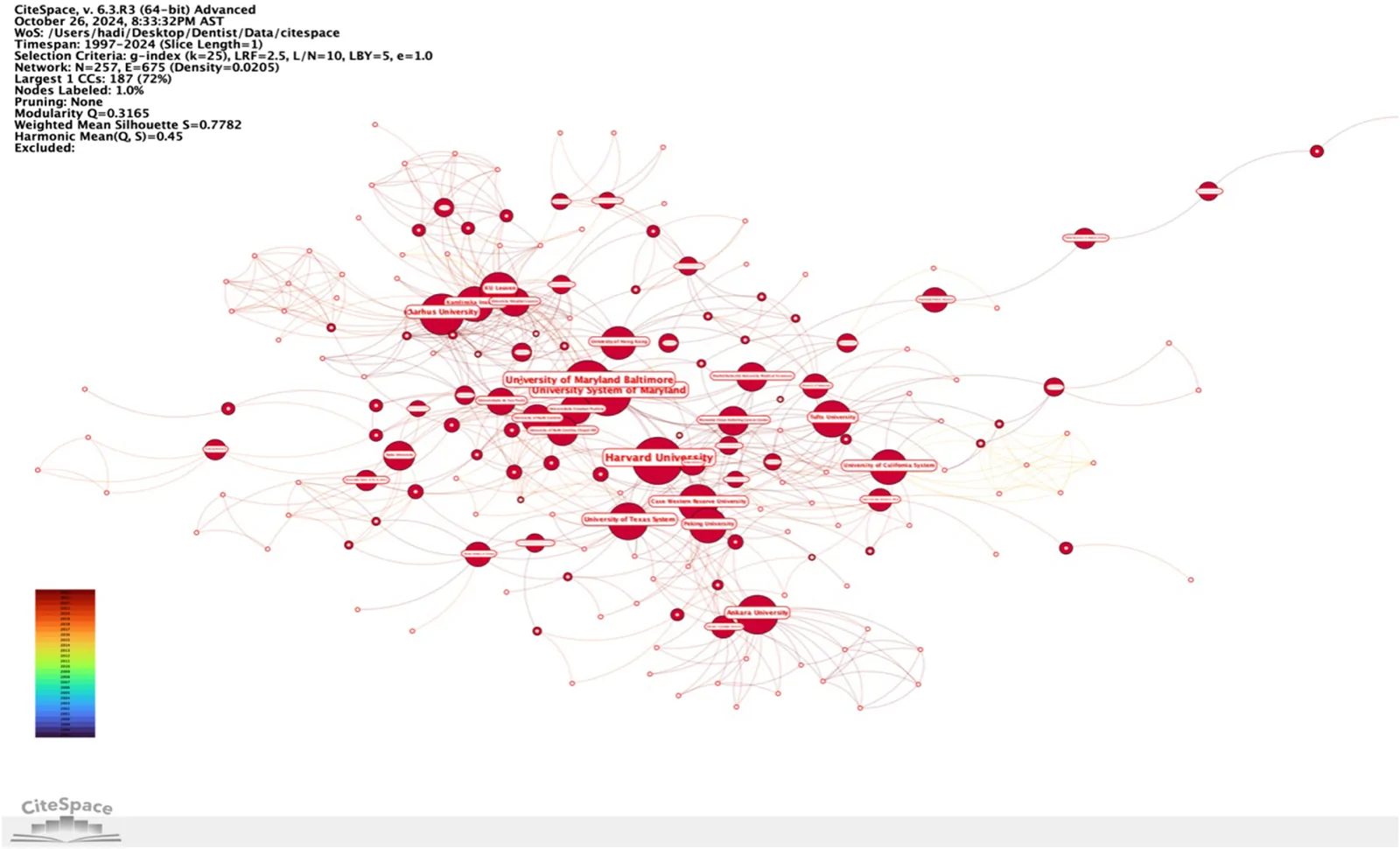
Institutions with the highest centrality in AI-related dental imaging research. The chart shows the leading institutions that play pivotal roles in collaborative networks.

**Figure 7.**
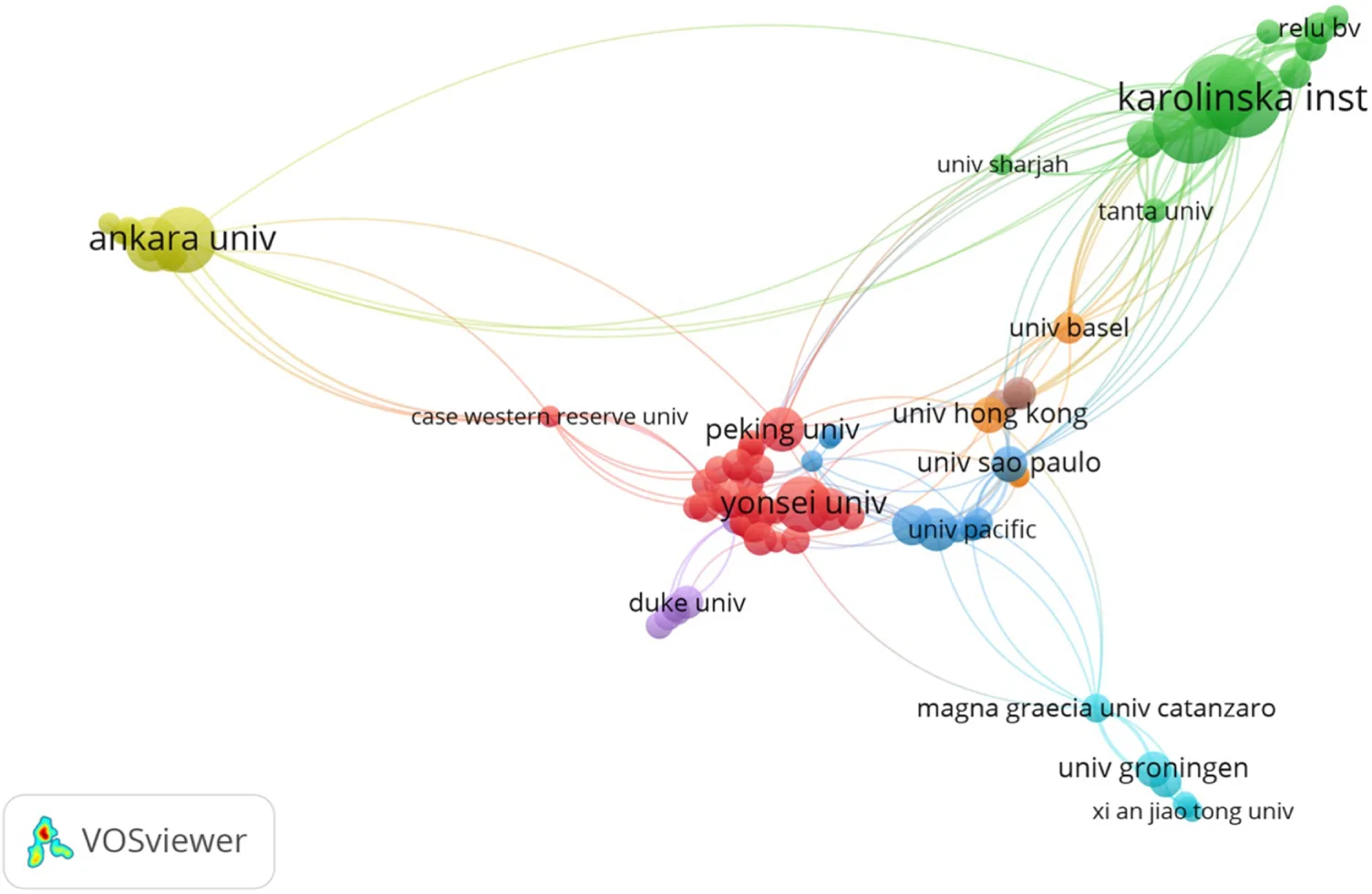
Network visualization of institutional collaboration in the field of artificial intelligence in dental imaging. This figure demonstrates the interconnectivity and cooperation among major research institutions.

In terms of institutional output, KU Leuven led with 46 publications, followed by University Hospital Leuven and Karolinska Institutet, each contributing 40 publications. Ankara University produced 30 publications, while Yonsei University, Peking University, and Eskisehir Osmangazi University each contributed 22. The University of Texas System followed with 21 publications, and both Yonsei University Health System and the Egyptian Knowledge Bank contributed 18 publications each (Table 3 and Fig. 6). Figure 7 illustrates the collaboration strength among different institutions.

**Table 3:** Top 10 institutions by number of publications.

Rank	Institutions	Number of publications
1	KU Leuven	46
2	University Hospital Leuven	40
3	Karolinska Institutet	40
4	Ankara University	30
5	Yonsei University	22
6	Peking University	22
7	Eskisehir Osmangazi University	22
8	University of Texas System	21
9	Yonsei University Health System	18
10	Egyptian Knowledge Bank	18

### Journals and co-cited journals

**Figure 8.**
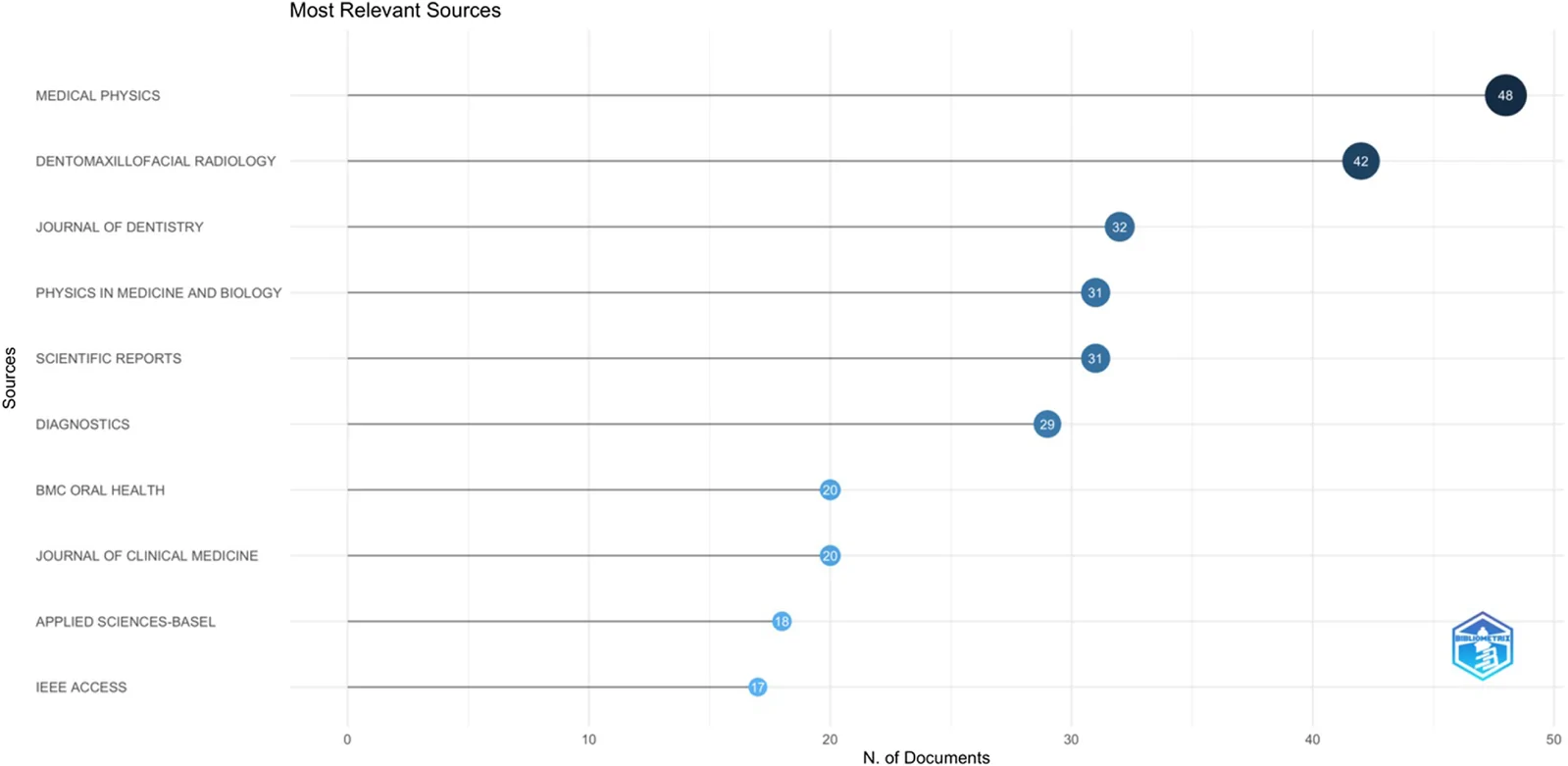
Top 10 journals publishing research on artificial intelligence in dental imaging. The chart ranks journals by the number of published articles, indicating the most active publication venues in the field.

The bibliometric analysis identified 171 journals publishing on AI in dental imaging. The Journal of Medical Physics led the way with 48 publications, followed by the Journal of Dentomaxillofacial Radiology with 42 and the Journal of Dentistry with 32. Other leading journals included Physics in Medicine and Biology and Scientific Reports, with 31 publications each, while Diagnostics contributed 29 publications. Additional significant sources were BMC Oral Health and the Journal of Clinical Medicine (20 publications each), Applied Sciences-Basel with 18, and IEEE Access with 17. Figure 8 highlights the top 10 journals in this area of AI in dental imaging.

**Figure 9.**
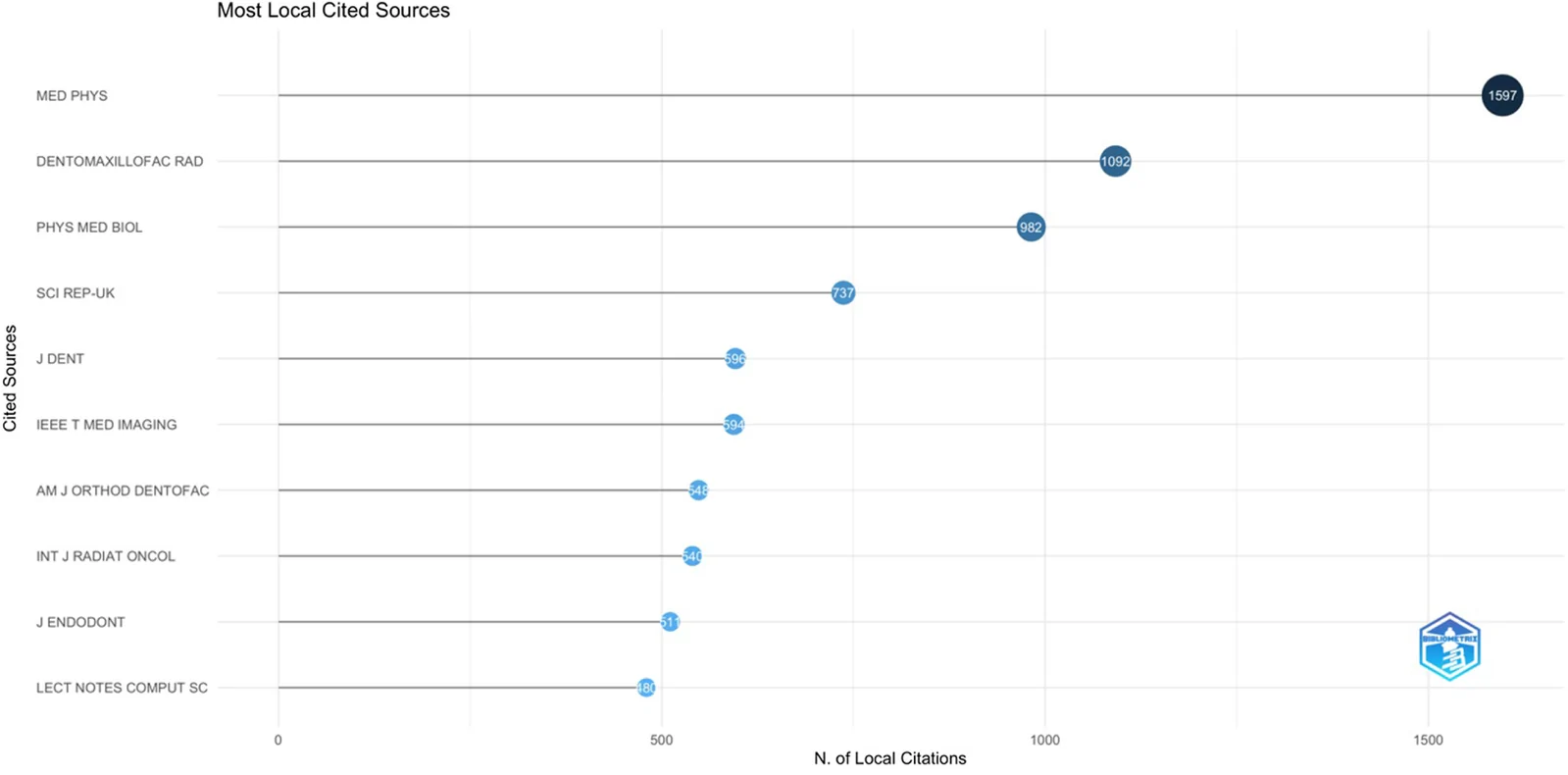
Top 10 journals publishing research on top 10 co-cited journals in artificial intelligence and dental imaging research. This figure displays the journals most frequently cited together, suggesting their influence and relevance in intelligence and dental imaging. The chart ranks journals by the number of published articles, indicating the most active publication venues in the field.

The co-citation analysis revealed that Medical Physics had the highest number of citations, with 1597. This was followed by Dentomaxillofacial Radiology with 1092 citations and Physics in Medicine and Biology with 982 citations. Scientific Reports received 737 citations, while the Journal of Dentistry garnered 596 citations. These represent the top five co-cited journals in the field of AI in dental imaging, as shown in Figure 9.

**Figure 10.**
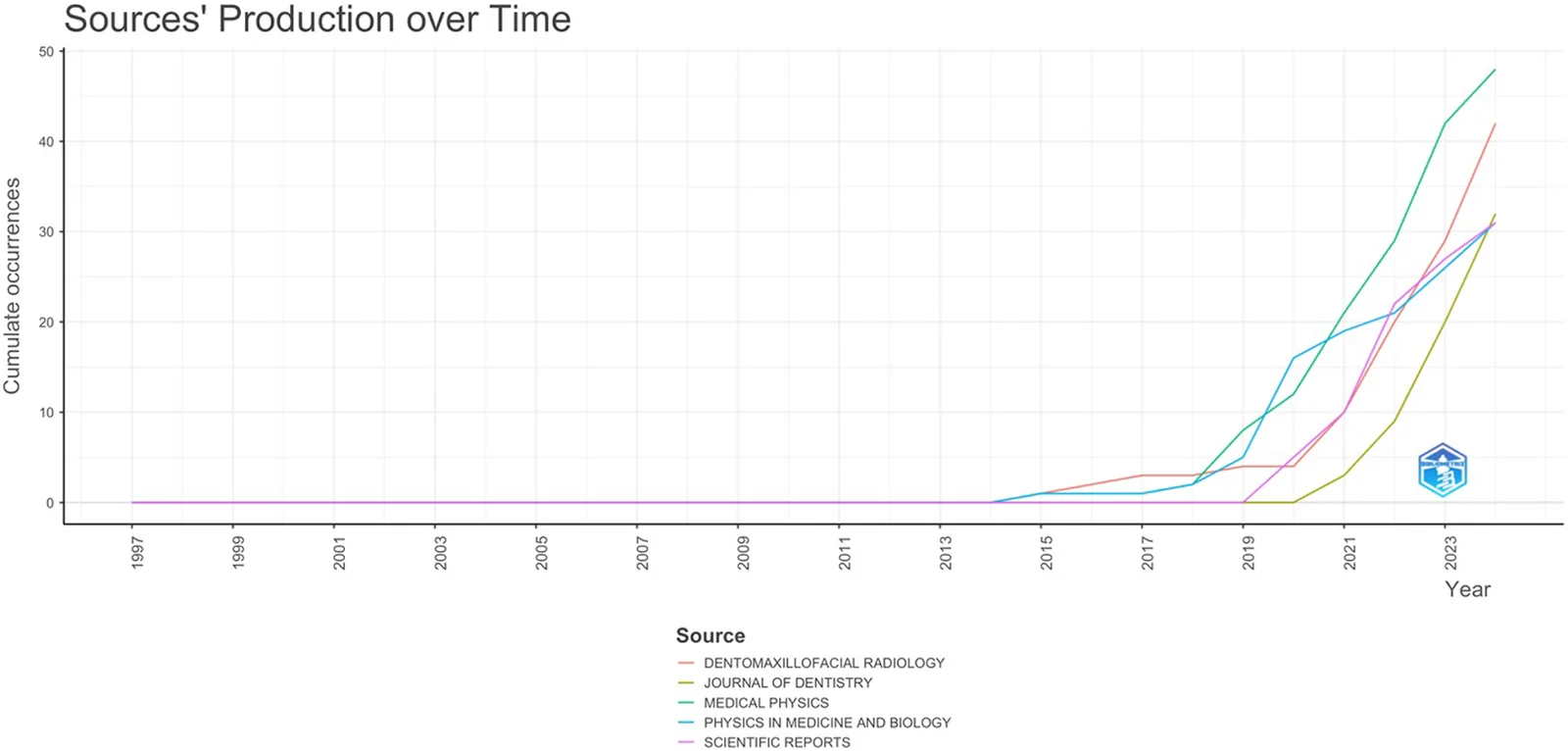
Publication trends over time across five leading journals in AI-based dental imaging. This timeline tracks the evolution of publication activity in key journals from 1997 to 2024.

The analysis of publication trends over time, as depicted in the provided data (Fig. 10), underscores the evolving research output across five key journals. From 1997 to 2014, there was no notable research activity, with all journals reporting zero publications. However, beginning in 2015, we observe a slow yet steady increase in publications. Journals such as Medical Physics, Dentomaxillofacial Radiology, and Physics in Medicine and Biology began contributing modestly, with minimal research output across the board.

A notable acceleration in publication volume began in 2019, particularly in Medical Physics and Physics in Medicine and Biology, which saw significant increases in the number of published articles. By 2020, Scientific Reports also began contributing to the trend, albeit on a smaller scale. The sharpest growth occurred from 2021 onward, with Medical Physics leading in total research output, publishing 48 papers by 2024, followed by Dentomaxillofacial Radiology and Physics in Medicine and Biology, which reached 42 and 31 publications, respectively.

Overall, this data reveal a sharp rise in research contributions in recent years, with an especially marked surge from 2020 onward. This reflects growing interest and advancements in these fields, particularly in medical and dental imaging, as seen in the dramatic uptick in output across all journals.

### Authors and co-cited authors

The analysis of contributions to the field of AI in dental imaging highlights several influential authors who have significantly shaped the research landscape. Reinhilde Jacobs emerged as the leading author, with the highest number of publications^[^39^]^ and an impressive total of 918 citations. Following Jacobs, Kaan Orhan published^[^28^]^ papers and accumulated 448 citations, while Ibrahim Sevki Bayrakdar contributed^[^21^]^ publications. Ozer Celik and Holger Willems rounded out the top five authors, with^[^17^]^ and^[^14^]^ publications, respectively (Table 4).

**Table 4:** Top 10 authors by publications, citations, and co-citations.

Number	Author with high number of publications	Number of publications	Author with high number of citations	Number of citations	The most co-cited authors	Number of co-citations
1	Jacobs, Reinhilde	40	Jacobs, Reinhilde	918	Lee, J.H.	234
2	Orhan, Kaan	28	Katsumata, Akitoshi	678	Schwendicke, F.	171
3	Bayrakdar, Ibrahim Sevki	21	Willems, Holger	664	Ronneberger, O.	141
4	Celik, Ozer	17	Fujita, Hiroshi	656	Hung, K.F.	105
5	Willems, Holger	14	Van Gerven, Adriaan	614	He, K.M.	94
6	Van Gerven, Adriaan	14	Ariji, Yoshiko	498	Lahoud, P.	91
7	Shujaat, Sohaib	14	Fukuda, Motoki	498	Orhan, K.	84
8	Bilgir, Elif	12	Ariji, Eiichiro	498	Zhang, Y.	81
9	Katsumata, Akitoshi	10	Orhan, Kaan	448	Tuzoff, D.V.	73
10	Ariji, Yoshiko	9	Seco, Joao	378	Pauwels, R.	72

In terms of citations, Reinhilde Jacobs led with 918 citations, a significant margin ahead of Kaan Orhan, who garnered 448 citations. Other prominent authors in citation counts include Akitoshi Katsumata (678 citations), Hiroshi Fujita (656 citations), and Holger Willems (664 citations), underscoring their substantial influence in the field (Table 4).

The analysis also identifies the most co-cited authors, with J.H. Lee leading the list with 234 co-citations. Other key co-cited authors include F. Schwendicke with 171 co-citations, O. Ronneberger with 141 co-citations, and K.F Hung. with 105 co-citations (Table 4).

These data underscore the significant impact of these researchers on advancing the field of AI in dental imaging. The influential work of these authors is reflected not only in their publications but also in their high citation and co-citation counts, which highlight their essential roles in shaping the ongoing developments in this area.

### Top-cited papers

**Figure 11.**
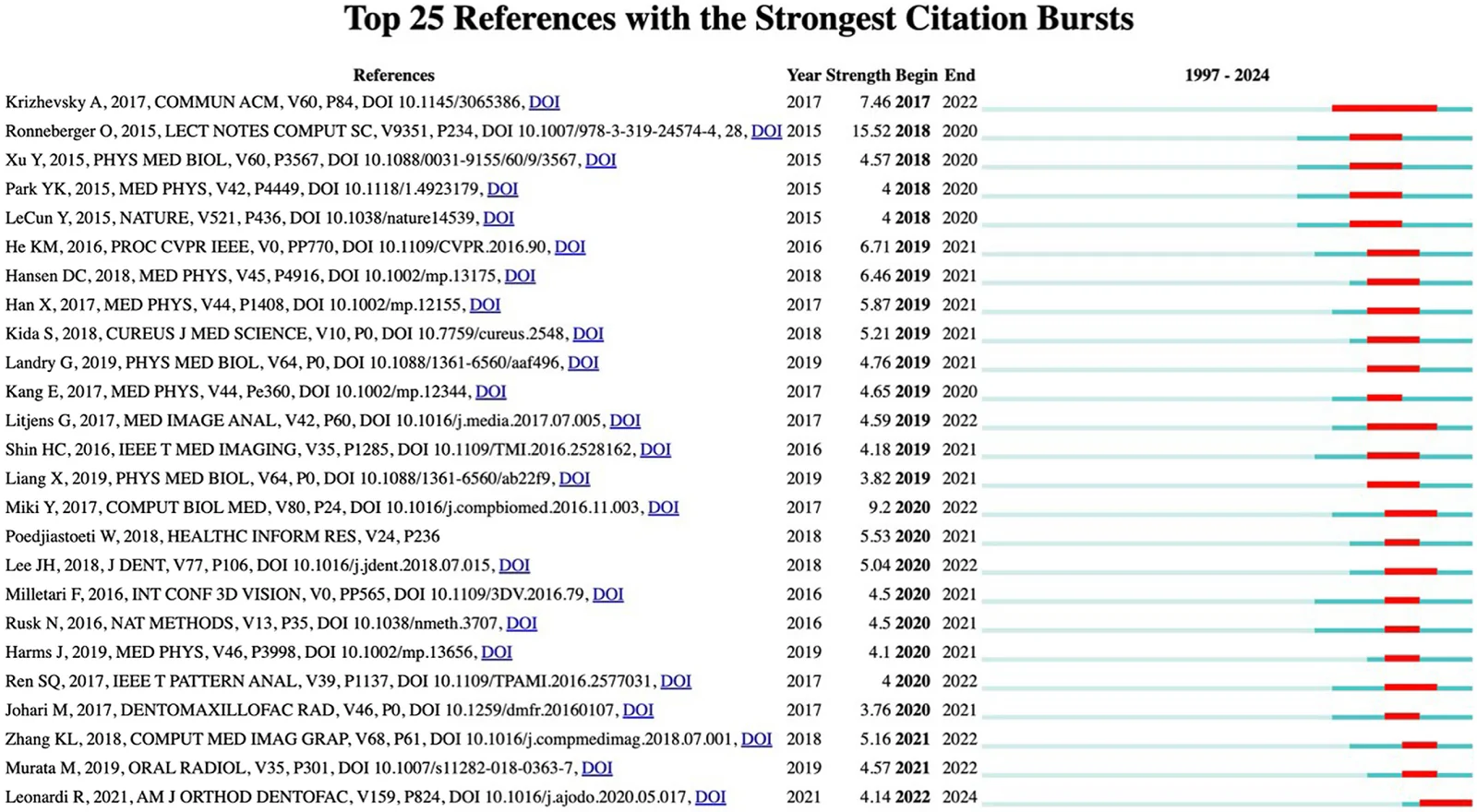
Citation burst analysis of key papers in artificial intelligence applied to dental imaging. This figure identifies papers that experienced a sudden surge in citations, indicating their emerging influence over time.

Table 5 presents the 10 most cited papers in the domain of AI applications in dental imaging, underscoring their substantial influence on the field. The paper with the highest citation count is “Tooth detection and numbering in panoramic radiographs using convolutional neural networks,” published in 2019, which has been cited 72 times. Following closely is the 2019 article “Deep Learning for the Radiographic Detection of Apical Lesions,” with 60 citations. The third most cited paper, published in 2018, is “Detection and diagnosis of dental caries using a deep learning-based convolutional neural network algorithm,” accumulating 54 citations. The fourth-ranked paper, also from 2019, is “A deep-learning artificial intelligence system for assessment of root morphology of the mandibular first molar on panoramic radiography,” which has received 49 citations. In the fifth place is “The use and performance of artificial intelligence applications in dental and maxillofacial radiology: A systematic review,” published in 2020, with 48 citations. Notably, Figure 11 highlights the citation bursts among the published papers in this area. A total of 25 papers experienced a notable surge in citations starting in 2017.

**Table 5:** Top 10 cited papers in the field of artificial intelligence in dental imaging.

Number	Title of most cited paper	Published year	Number of citations
1	Tooth detection and numbering in panoramic radiographs using convolutional neural networks	2019	72
2	Deep learning for the radiographic detection of apical lesions	2019	60
3	Detection and diagnosis of dental caries using a deep learning-based convolutional neural network algorithm	2018	54
4	A deep-learning artificial intelligence system for assessment of root morphology of the mandibular first molar on panoramic radiography	2019	49
5	The use and performance of artificial intelligence applications in dental and maxillofacial radiology: A systematic review	2020	48

### Keyword trends, hotspots, and cluster analysis

**Figure 12.**
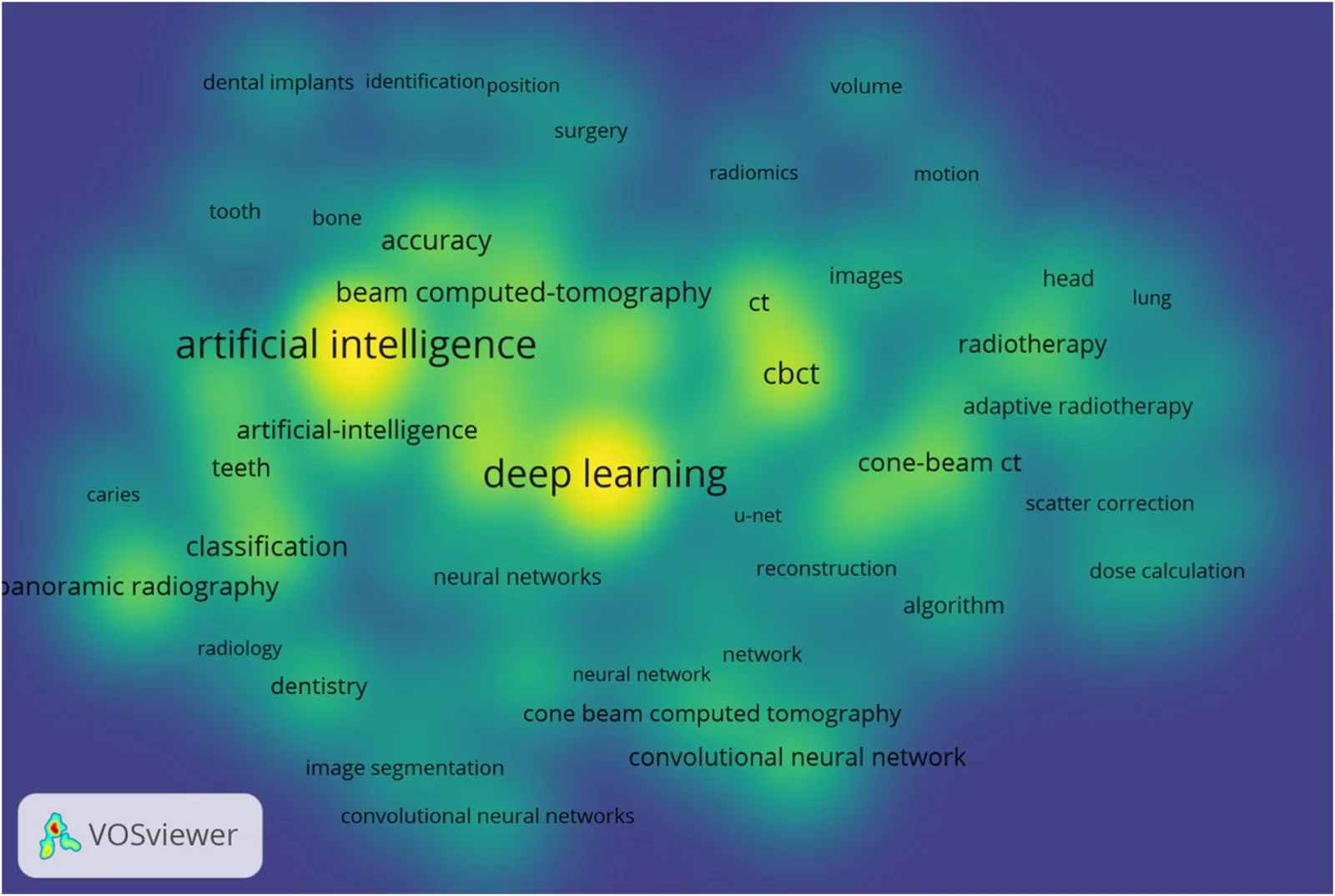
Density visualization of frequent keywords in AI-based dental and maxillofacial radiology. Warmer colors represent higher keyword frequencies, highlighting core research themes within the field.

The analysis of keywords revealed several terms frequently appearing in research focused on AI applications in dental imaging. The 10 most prominent keywords identified were “artificial intelligence” (*n* = 269), “deep learning” (*n* = 248), “CBCT” (*n* = 117), “machine learning” (*n* = 100), “cone-beam computed tomography” (*n* = 90), “beam computed-tomography” (*n* = 77), “classification” (*n* = 75), “accuracy” (*n* = 75), “CT” (*n* = 74), and “segmentation” (*n* = 74) (Fig. 12).

**Figure 13.**
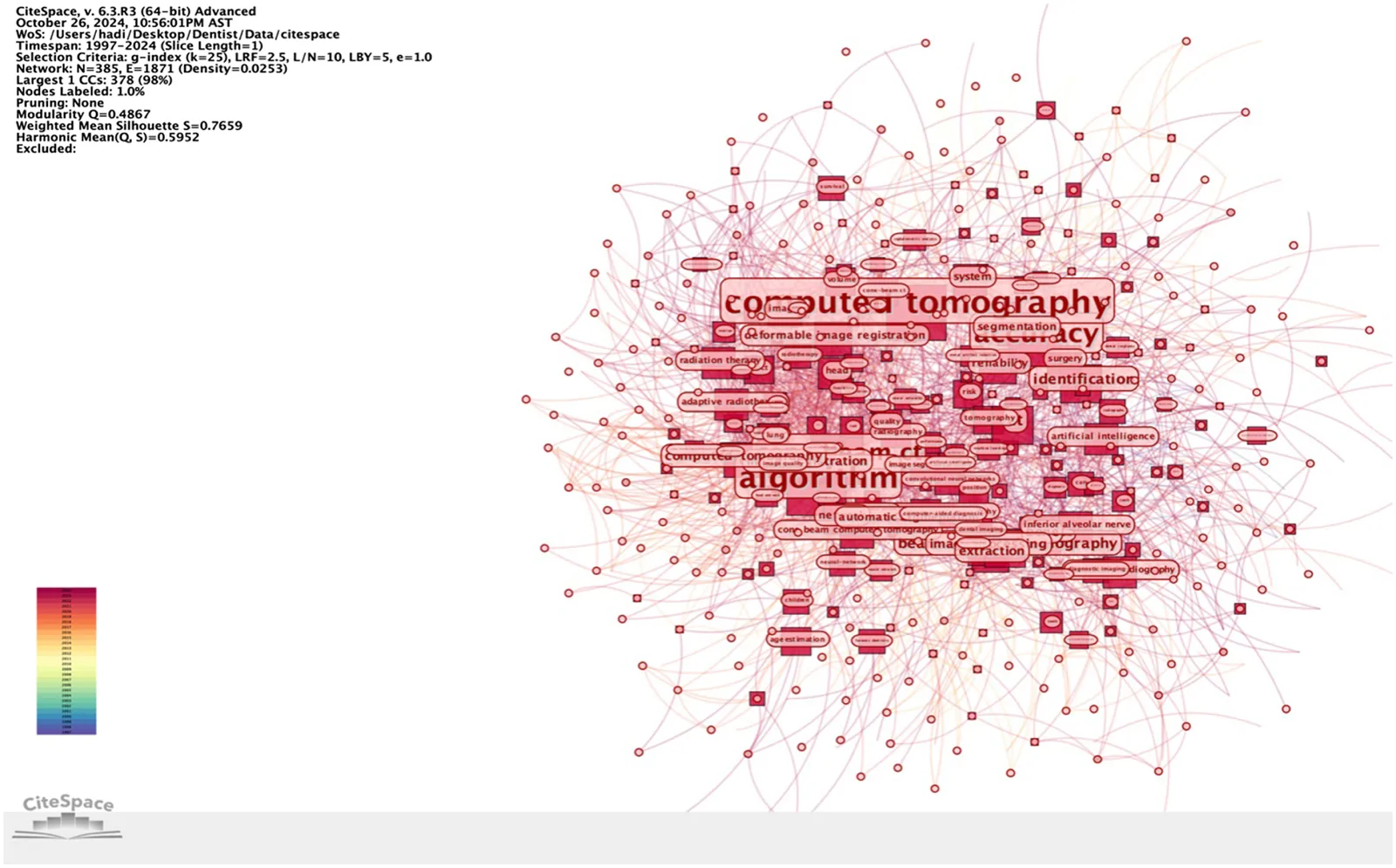
Keyword centrality map in artificial intelligence and dental imaging research. Keywords with higher centrality are more influential in connecting different research clusters.

The analysis of central keywords, highlighting the most significant terms within the research network, identified the following top 10: “computed tomography” (0.15), “algorithm” (0.15), “accuracy” (0.13), “cone beam CT” (0.11), “CT” (0.08), “beam computed tomography” (0.07), “bone” (0.07), “identification” (0.07), “image registration” (0.06), and “computed-tomography” (0.06) (Fig. 13).

**Figure 14.**
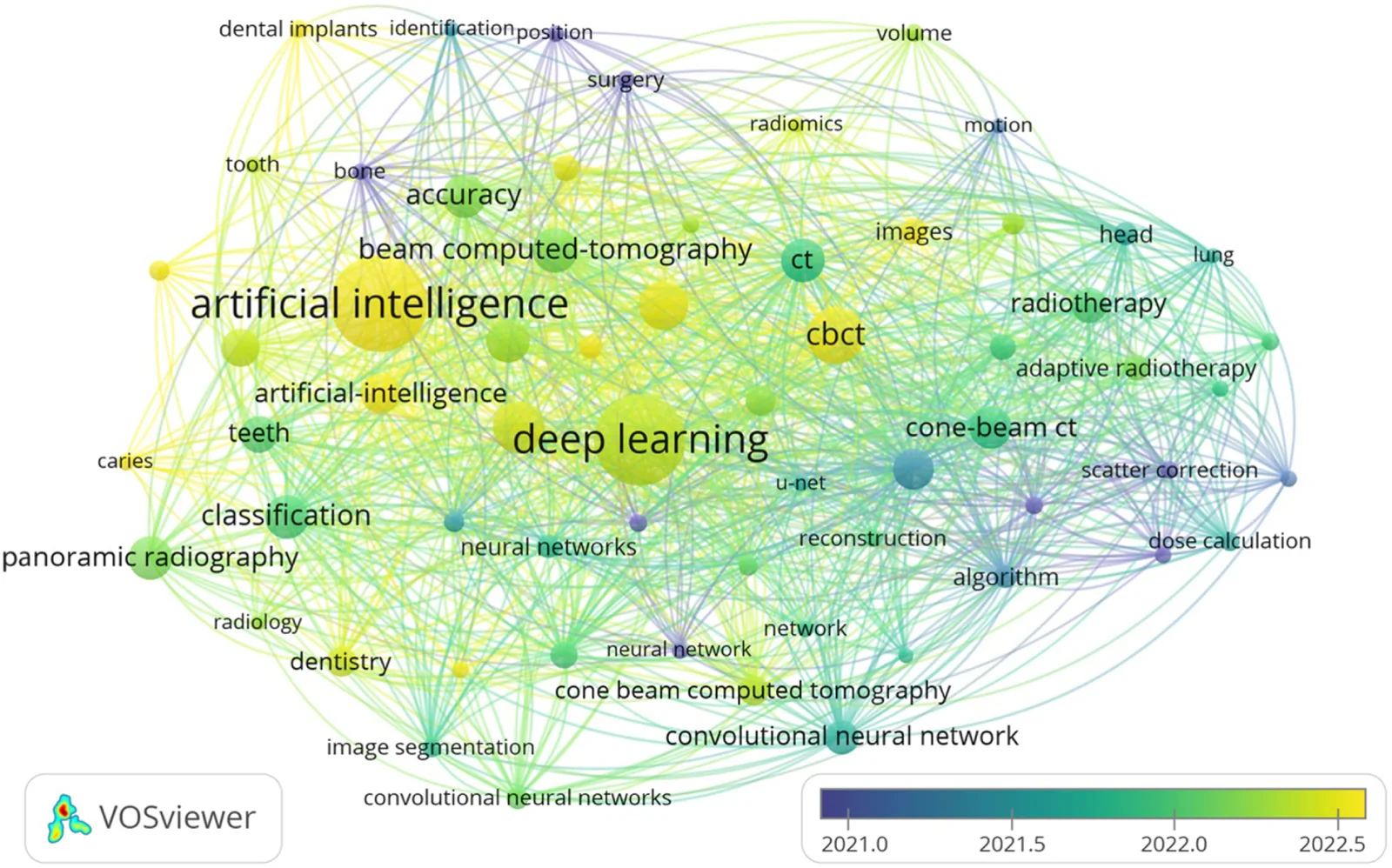
Overlay visualization of emerging keywords in AI-related dental imaging literature. The color gradient represents the temporal evolution of keywords, with more recent terms shown in lighter colors.

Our analysis identified the 10 most recent keywords emerging in the field of AI in dental imaging, highlighting the latest research trends and focal areas. These keywords include “artificial intelligence,” “deep learning,” “CBCT,” “machine learning,” “cone-beam computed tomography,” “beam computed-tomography,” “classification,” “accuracy,” “CT,” and “segmentation.” These terms emphasize key interests within the research community and suggest potential directions for future studies (Fig. 14).

**Figure 15.**
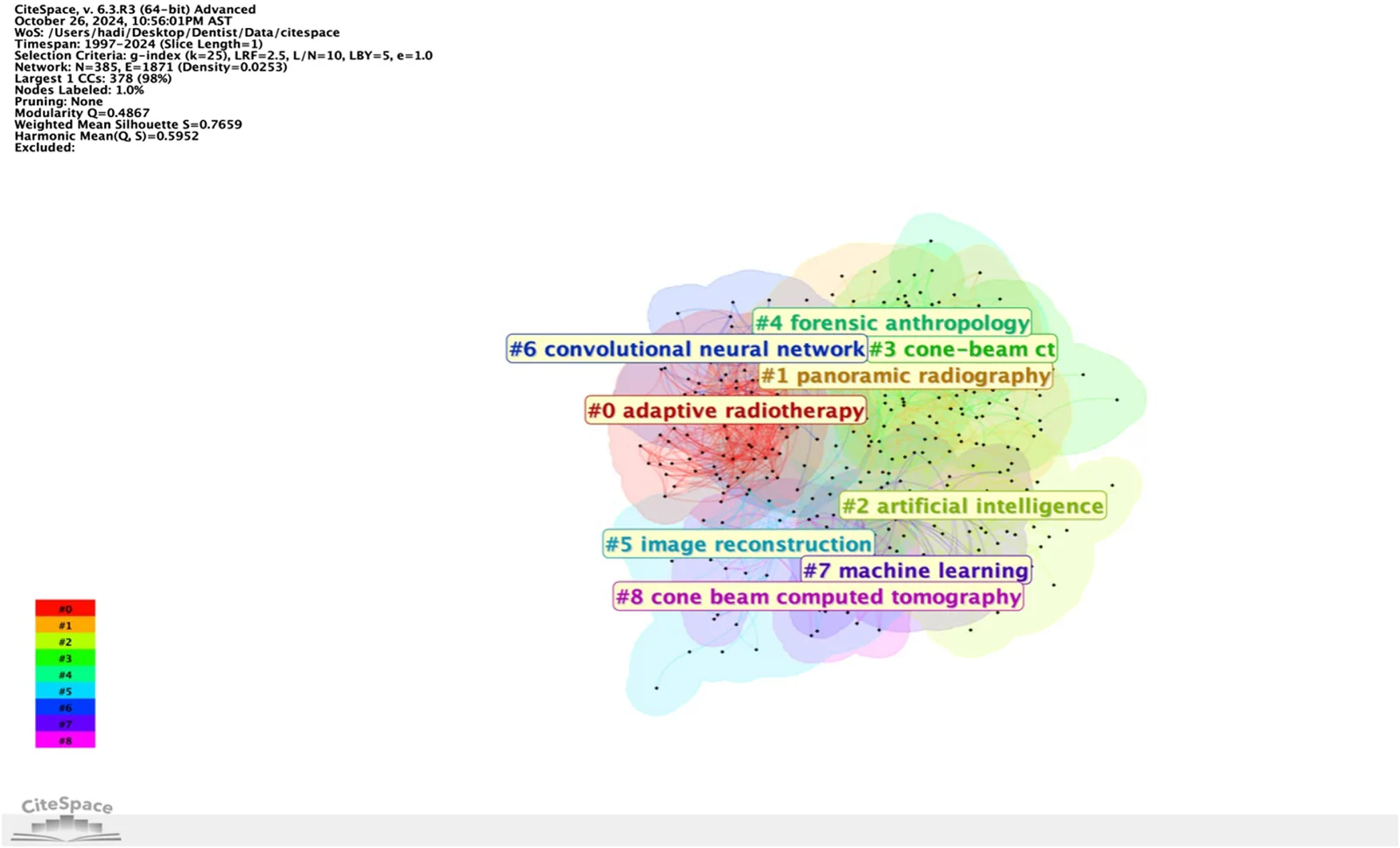
Cluster analysis of major research themes in artificial intelligence applied to dental imaging. This network map identifies distinct thematic areas through keyword clustering and co-occurrence relationships.

**Figure 16.**
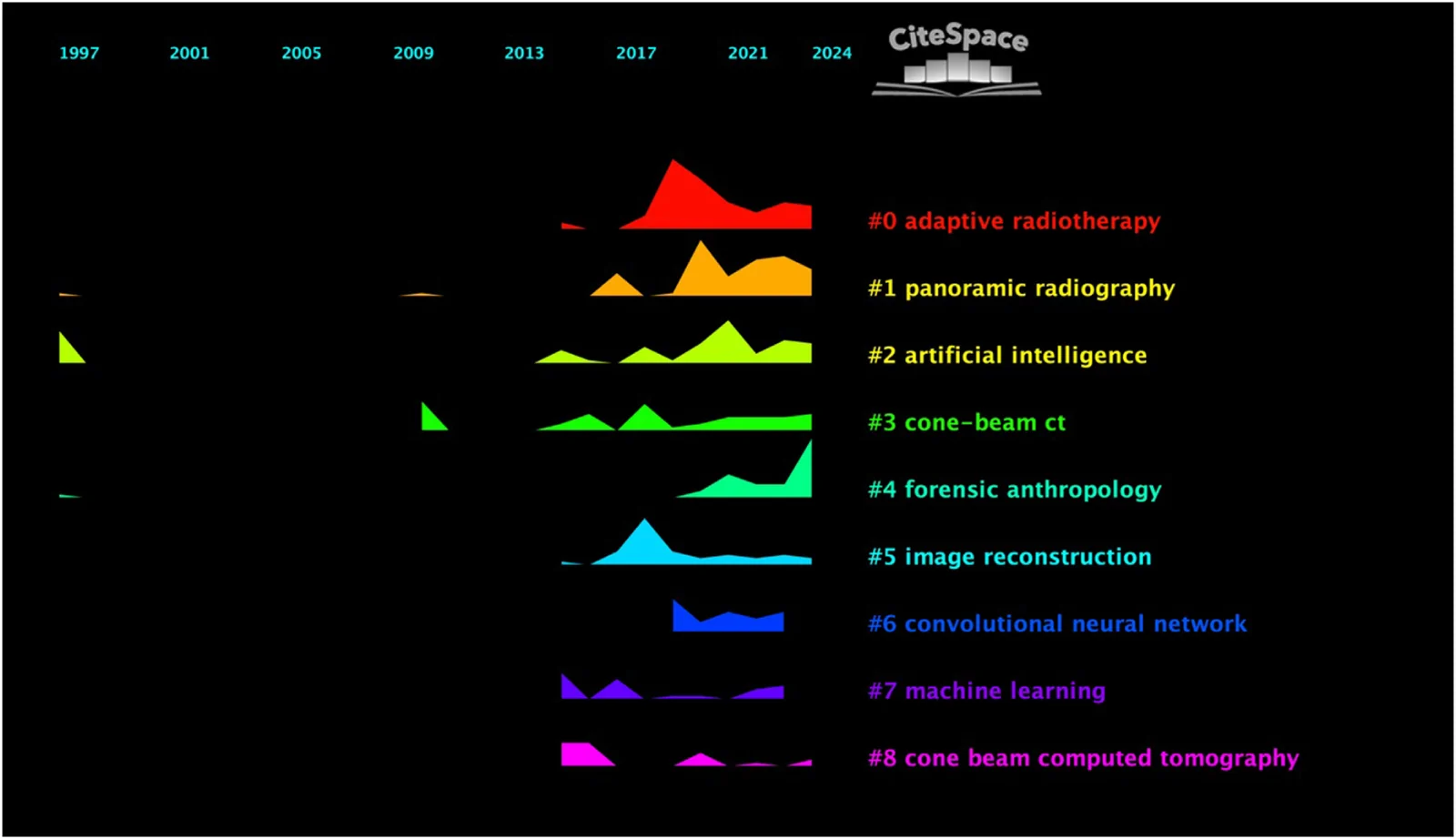
Time-trend visualization of research topics in AI-based dental and maxillofacial radiology. This timeline shows the chronological development of key clusters, revealing how the research focus has shifted over time.

The cluster analysis identified eight key clusters, highlighting significant areas of research focus. These clusters include Adaptive Radiotherapy (#0), Panoramic Radiography (#1), Artificial Intelligence (#2), Cone-Beam CT (#3), Forensic Anthropology (#4), Image Reconstruction (#5), Convolutional Neural Network (#6), Machine Learning (#7), and Cone-Beam Computed Tomography (#8) (Figs 15 and 16).

## Discussion

This bibliometric study underscores the growth of research on AI applications in dental and maxillofacial radiology, highlighting key trends over the past years. The marked increase in publications, particularly after 2016, indicates the rapid advancements in AI technologies and their growing application in dental and maxillofacial radiology. The surge from 11 publications in 2017 to 218 in 2024 reflects the field’s evolution from nascent explorations to an established area of academic and clinical interest. This accelerated research output aligns with broader global trends in healthcare AI investment and policy development, including increased national funding initiatives and recent regulatory approvals of AI systems for dental applications^[^40^]^.

The recent advancements in deep learning and imaging technologies have improved diagnostics and automation in dental practice. Among different radiographical modalities in the field of dental and maxillofacial radiology, CBCT and panoramic radiography are frequently applied for AI-assisted data analysis^[^39,41^]^. CBCT is indicated among the most prominent, central, and recent keywords, while panoramic radiography is among the most frequent topics in cluster analysis.

CBCT, as the customized form of the conventional CT technique with lower dose irradiation, is an X-ray imaging method providing a 3D grasp of the dental and maxillofacial structure^[^42^]^. This approach can assess the targeted area through different planes, thus avoiding anatomical superimpositions and providing a clear view of dental caries, lesions, traumas, and so on^[^42^]^. AI-enhanced CBCT can outperform other radiographic modalities. In this regard, Johari *et al* have indicated that the incorporation of AI in CBCT imaging can markedly enhance the accuracy, sensitivity, and specificity of CBCT in detecting vertical root fractures compared with AI-incorporated diagnosis in periapical radiographs, which is a 2D imaging modality^[^43^]^. Among the existing AI systems, CNN is one of the most well-known algorithms frequently applied in this field. In this regard, the CNN algorithm is indicated as a useful tool to provide a quantitative and qualitative assessment of the alveolar bone for implant insertion through analyzing CBCT modalities^[^44,45^]^. A subtype of CNN in image analysis is called U-Net. This CNN algorithm is shown to be a proper candidate to detect the inferior alveolar nerve canal to avoid any damage during implant surgeries^[^45,46^]^.

Aside from the mentioned applications, AI models in the CBCT modality are also used for the diagnosis and differentiation of tumors, including ameloblastoma and odontogenic keratocyst, using the deep learning Inception v3 algorithm^[^45,47^]^; the diagnosis of temporomandibular joint diseases using different algorithms, including the K-nearest neighbors algorithm^[^45,48^]^; and maxillary and mandibular segmentation using CNN and recurrent neural networks^[^45,48,49^]^. The precision and accuracy of CBCT compared to other radiographic modalities is the underlying reason for CBCT to be among the most prominent, central, and recent keywords in this field.

Another frequently used extraoral radiology method is panoramic radiography, which provides a scan of the whole maxillofacial hard tissue along with surrounding bone structures. Concerning the application of AI in panoramic radiography, Webster and Fraser have noted a significant surge in publications on this topic, with different rates of accuracy reported for tooth identification and numbering (93.67%), caries detection (91.5%), and diagnosis of osteoporosis (89.29%)^[^39^]^. Diagnosis of periodontal bone loss and maxillary sinusitis was also reported across the studies. Moreover, Zadrożny *et al* indicated that the application of AI in diagnosing pathologies and anatomical structures in panoramic radiography has overall higher specificity (0.847–1.000) than sensitivity (0.390–0.961)^[^50^]^. The highest specificity was observed for underfilled and overfilled root canals (1.000), while the highest sensitivity was noted for the detection of missing teeth (0.961) and prosthetic restorations (0.957). Detection of periodontal bone loss and periapical lesions exhibited the lowest specificity (0.847) and sensitivity (0.390), respectively. It is indicated that the location of the periapical lesions in panoramic radiography is a decisive factor for accurate diagnosis, as the most easily identified lesions were located in the mandibular canine, premolar, and molar areas, while the lowest accuracy was associated with the maxillary molars and maxillary/mandibular incisor areas^[^51^]^. These key points should be considered when AI is implemented in the interpretation of pathologies in panoramic radiographs. However, due to the limitations in panoramic radiography, including superimpositions and size distortions, AI-enhanced panoramic radiography may require further improvements^[^52^]^. The current bibliometric investigation indicates that despite the higher frequency of panoramic radiography in cluster analysis and its application in the top 10 cited papers in the field, CBCT is more noticeable among the most common keywords and the keywords with centrality among the published studies. This suggests a possible shift in attention toward the application of AI from panoramic radiography to CBCT modality. Nevertheless, since panoramic radiography produces less radiation exposure, its application instead of CBCT is still justified when no specific indication for CBCT is noted.

Among the top 10 cited papers in the field of AI in dental and maxillofacial radiology, seven studies applied CNN systems for tooth detection and numbering^[^53^]^, detection of apical lesions^[^54,55^]^, dental caries^[^56^]^, presence of extra roots on the distal root of the mandibular first molar^[^57^]^, periodontal bone loss^[^58^]^, and vertical root fracture^[^59^]^. Most of the studies were conducted on panoramic radiographs, while two studies used CBCT^[^55,57^]^ and one study used periapical radiography^[^56^]^. All of the studies reported favorable outcomes concerning the accuracy, sensitivity, and specificity of the CNN systems in the segmentation and classification of teeth and dental pathologies. Among the top-cited papers in this field, three studies were systematic^[^22^]^, narrative^[^60^]^, and scoping reviews^[^61^]^.

The bibliometric analysis indicated significant global contributions, with the United States and China being the greatest sources of publications. These countries also exhibit high centrality within collaborative networks, underscoring their roles as key hubs in advancing the field. The involvement of other countries, such as South Korea, Germany, and Belgium, further emphasizes the global nature of research efforts.

Concerning the leading institutions in this field, KU Leuven, University Hospital Leuven, and Karolinska Institutet have emerged as top contributors, reflecting their strategic focus on integrating AI into dental research. The dominance of journals, including *Medical Physics* and *Dentomaxillofacial Radiology,* highlights the multidisciplinary nature of the field, bridging dental and maxillofacial radiology with computer sciences. These journals have also played a critical role in shaping research priorities by emphasizing topics, such as tooth identification, pathology diagnosis by CNN systems via proper segmentation, and classification methods.

The bibliometric findings of this study provide important context for the current limitations of AI applications in dental and maxillofacial radiology. The dominance of deep learning and convolutional neural network–based approaches, as reflected by keyword frequency and highly cited publications, underscores the field’s focus on algorithm development and diagnostic performance. However, the concentration of research on a limited number of imaging modalities, particularly CBCT and panoramic radiography, and the reliance on relatively homogeneous datasets may partly explain persistent challenges related to model generalizability and external validation.

Furthermore, the high citation impact of studies emphasizing accuracy and segmentation highlights the prioritization of performance metrics, while comparatively fewer publications address interpretability, clinical transparency, and real-world implementation. This imbalance, revealed through co-citation and keyword analyses, aligns with ongoing concerns regarding the “black-box” nature of AI systems and their limited integration into routine clinical workflows. The bibliometric evidence, therefore, suggests that current AI limitations are not solely technical but are also shaped by prevailing research priorities within the field.

Although the current advancements in AI systems have brought about noticeable prospects in dental and maxillofacial radiology, this process is still in its initial stages. Clinicians and researchers are required to provide valuable insights into designing the future path this field of research can take. In order to chart this path, clinicians and researchers should consider the current shortcomings, needs, and the future conditions that patients may encounter in radiology clinics. Moreover, a proper understanding of AI systems and their abilities, along with the shortcomings of every deep learning algorithm, can enable clinicians to participate in developing custom-developed AI systems specific to radiology with desirable characteristics.

A closer cooperation between radiologists and computer scientists in the step-by-step development of AI models can be taken more seriously in the near future^[^61,62^]^. Providing new solutions and protocols for improving generalizability and avoiding model overfitting is essential. This issue should be considered both at the level of AI system development and during the training phase, in terms of the number of training samples and how closely each sample is close to the common characteristics of the disease. Another issue in this regard is the variation in the manifestation of the disease based on personal, geographical, and other possible factors that affect diagnosis and potentially limit accuracy. Aside from including additional data on patient characteristics to address generalizability issues, introducing a minimum level of acceptable accuracy, sensitivity, and specificity can be of high significance for future research.

In order for AI systems to be used in daily clinical practice, providing clinicians with the ability to monitor the mechanisms of AI systems' processing and interpretability is critical^[^63^]^. This can eliminate potential misunderstandings and the ineffectiveness of cooperation between clinicians and AI systems in the future. It can also address possible legal issues on the disparities in clinical judgment between clinicians and AI systems. Moreover, in cases where AI outperforms clinicians in diagnosis, the interpretability of AI systems can enlighten clinicians on possible new signs and associations for better diagnosis. Therefore, clinicians can also benefit from the possible new insights in this regard.

Another issue to consider for the future direction of research in this regard is understanding the cognitive characteristics of AI models^[^64^]^. Currently, AI systems seem to exhibit an authoritative voice when reporting outcomes or providing differential diagnoses ^[^65^]^. Self-awareness of the possibility of providing false judgment is a critical issue in this matter, as well as understanding the difference between differential and absolute diagnosis when dealing with medical and radiological issues, which can have consequences on health status, moral, and legal matters. Therefore, the outcomes provided by AI systems should be considered with caution and supervised by an expert radiologist.

The increasing application of AI in dental and maxillofacial radiology has important clinical and public health implications that extend beyond technological innovation. AI-assisted diagnostic systems have demonstrated the potential to improve diagnostic accuracy, reduce inter- and intra-observer variability, and enhance workflow efficiency, thereby supporting more consistent, timely, and standardized patient care. From a broader public health perspective, AI-driven radiological tools may facilitate teleradiology services and expand access to specialized diagnostic expertise in underserved or resource-limited regions, where shortages of trained radiologists remain a persistent challenge^[^5,66,67^]^. Notably, the high citation impact of studies focusing on clinically relevant applications reflects the growing translational orientation of AI research and its increasing alignment with real-world clinical demands. At the same time, these trends underscore ongoing challenges related to clinical validation, generalizability across diverse populations and imaging protocols, and ethical considerations in AI deployment^[^68–70^]^. Collectively, the bibliometric evidence suggests that AI is increasingly positioned as a complementary tool rather than a replacement for clinicians, with the potential to promote diagnostic equity, support evidence-based decision-making, and optimize treatment planning in dental and maxillofacial radiology.

### Limitations

This bibliometric study provides a comprehensive overview of research trends, influential contributors, and thematic developments in AI-assisted dental and maxillofacial radiology. Nevertheless, several inherent limitations of bibliometric analyses should be considered when interpreting the findings. First, the rapid evolution of AI research is accompanied by continuously emerging terminologies and methodological innovations. Despite the use of a comprehensive and carefully designed search strategy, relevant studies employing novel or unconventional terms may not have been fully captured. This limitation is common in bibliometric studies of fast-growing interdisciplinary fields and may influence the completeness of the dataset. Second, this analysis relied exclusively on the Web of Science Core Collection database. Although this database is widely recognized for its high-quality indexing and broad journal coverage, it does not include all journals, conference proceedings, or regional publications. Consequently, relevant AI-related studies indexed in other databases, such as Scopus, PubMed, or IEEE Xplore, may have been omitted, potentially affecting the representation of certain research communities or geographic regions. Third, publication and citation biases are intrinsic to bibliometric studies. Highly cited articles, established journals, and well-known institutions tend to receive greater visibility, which may result in the underrepresentation of recent studies, emerging researchers, or innovative AI applications from less prominent institutions. In addition, the exclusion of non-English publications may have limited the inclusion of valuable contributions from non-English-speaking regions. This language restriction should be acknowledged as a potential source of bias, as it may affect the geographic and institutional representation within the dataset and could underrepresent research output from regions where English is not the primary language of scientific publication. Fourth, bibliometric analyses do not allow for direct evaluation of the scientific quality, methodological rigor, or clinical validity of the included publications. The analysis focuses on quantitative indicators such as publication counts, citations, and network relationships, rather than on the critical appraisal of study design, data quality, or outcome reliability. Therefore, highly cited AI studies may not necessarily represent the highest scientific or clinical quality. Finally, bibliometric analyses are inherently descriptive and retrospective. While they are effective for identifying publication patterns, collaboration networks, and research hotspots, they cannot establish causal relationships or predict future developments in AI-based dental and maxillofacial radiology with certainty. The findings therefore represent a snapshot of the research landscape based on data available at the time of analysis. Despite these limitations, this study provides valuable insights into the global research structure and evolving trends of AI applications in dental and maxillofacial radiology. Awareness of these limitations enables a more cautious interpretation of the results and underscores the need for complementary qualitative and systematic reviews in future research.

## Conclusion

Based on the findings from our bibliometric analysis, several key conclusions emerge regarding the landscape of AI applications in dental and maxillofacial radiology. First, there has been a significant and accelerating growth in research output since 2016, reflecting the expanding interest in the utilization of AI to enhance diagnostic precision and clinical decision-making in this specialized field. The United States leads as the most prolific contributor, demonstrating robust international collaboration, followed closely by China and South Korea. Influential institutions such as KU Leuven and University Hospital Leuven play pivotal roles in advancing this research area.

Journals of Medical Physics and Dentomaxillofacial Radiology have become central to disseminating groundbreaking work, illustrating the growing academic interest and the rising impact of AI-driven innovations in radiology. The current research landscape is dominated by the development and application of deep learning and machine learning algorithms, particularly for tasks such as image classification, segmentation, and anomaly detection. Emerging trends highlight automated quantification, the integration of AI into clinical workflows, and personalized treatment strategies.

The current advancements demonstrate the potential of AI-enhanced radiology in identifying teeth and detecting dental caries, periodontal bone loss, and periapical lesions. In addition, AI can contribute to the differential diagnosis of pathologic lesions. In all these areas, AI can play an adjunct role, enhancing clinical diagnosis and optimizing treatment planning.

Despite these advancements, challenges remain, particularly in translational research. Future research must address current limitations by fostering closer collaboration between clinicians and computer scientists to develop tailored, reliable, and ethically sound AI systems. Future bibliometric studies will also be valuable for tracking the emergence and impact of novel AI approaches as these technologies mature and become more prominent in the literature. This multidisciplinary effort will be essential to fully utilize AI’s potential to revolutionize dental and maxillofacial radiology and improve patient outcomes.

## Data Availability

Data from the study can be provided by the corresponding author upon reasonable request.

## Research Registration Unique Identification Number (UIN)

The study is a bibliometric study, and UIN is not applicable.
